# Assessment of Blood Parameters in Free-Ranging Red Deer (*Cervus elaphus*) from the Eastern Carpathians Between Autumn and Early Winter

**DOI:** 10.3390/vetsci12090915

**Published:** 2025-09-19

**Authors:** Mircea Lazăr, Răzvan Mihail Radu-Rusu, Ioana Acornicesei, Roxana Lazăr

**Affiliations:** 1 Department of Preclinics, Faculty of Veterinary Medicine, Iasi University of Life Sciences Ion Ionescu de la Brad, 700489 Iasi, Romania; mircea.lazar@iuls.ro; 2 Department of Control, Expertise and Services, Faculty of Food and Animal Sciences, “Ion Ionescu de la Brad” University of Life Sciences, 700489 Iasi, Romania

**Keywords:** *Cervus elaphus*, wildlife physiology, wildlife health, hematology, serum biochemistry

## Abstract

**Simple Summary:**

Red deer (*Cervus elaphus*) are one of the most important wild species in European forests. These animals experience natural changes in behavior and body function throughout the year. In autumn, male deer go through a demanding mating period called the rut, followed by winter, when food is scarce and temperatures are low. These seasonal challenges may affect their blood and health indicators. In this study, we examined blood samples from 40 adult red deer hunted legally in the Eastern Carpathian Mountains of Romania. We compared males and females, both in autumn and in winter, to see how their bodies adapt. We analyzed baseline hematological and biochemical parameters, including red blood cell count (RBC), hemoglobin (HGB), hematocrit (HCT), white blood cell (WBC) count, platelets (PLTs), cholesterol, glucose, and serum proteins. Our results showed that males had significantly higher HGB and HCT values during and after the rut, while females maintained more stable values across seasons. RBC followed the same seasonal pattern, but the difference between sexes did not reach statistical significance. These differences are linked to seasonal behavior and energy needs. This research helps us understand how red deer stay healthy in the wild and provides useful reference data for wildlife managers and conservation efforts.

**Abstract:**

Understanding physiological variability in wild ungulates is essential for ecological monitoring and sustainable wildlife management. This study aimed to examine whether sex and season (autumn vs. early winter) significantly influence hematological and biochemical parameters in free-ranging red deer (*Cervus elaphus*) from the Eastern Carpathians, Romania. A total of 40 legally harvested adult individuals (20 males, 20 females) were included, and blood samples were collected post-mortem under standardized conditions to minimize pre-analytical variability. Hematological parameters (WBC, RBC, HGB, HCT, PLTs) and serum biochemical markers (glucose, urea, total cholesterol, triglycerides, total protein) were analyzed using automated veterinary analyzers. Statistically significant sex-related differences were found in hematocrit during autumn and hemoglobin concentration during winter, with higher values in males. Seasonal variation within sex groups was not significant but indicated a physiological trend toward hemoconcentration in winter. Biochemical values remained within reference ranges and showed no significant differences across groups. Pearson’s correlation analysis revealed a strong association between hematocrit and urea, and moderate correlations were observed between WBC and glucose, suggesting links between oxygen transport, protein metabolism, and energy balance. Environmental factors such as reduced food availability and temperature shifts during winter likely contribute to these physiological adjustments. These results provide baseline data for the physiological assessment of red deer populations and support the development of ecological health indicators in wildlife monitoring programs. Future studies incorporating hormonal and immunological biomarkers across multiple seasons are encouraged to further understand adaptive responses in cervids.

## 1. Introduction

The red deer (*Cervus elaphus*) is one of the most widespread and ecologically adaptable cervids in Europe, holding both ecological and cultural importance [1,2]. Its remarkable plasticity enables survival in highly diverse habitats, from dense forests to open grasslands [3,4,5]. Nevertheless, population dynamics are uneven across the continent: while some Central European populations experience genetic erosion and demographic decline due to habitat fragmentation and anthropogenic pressures, Romanian populations remain relatively stable, with more than 35,000 individuals distributed mainly in the Carpathian Mountains [6,7,8]. This regional heterogeneity emphasizes the importance of conducting population-specific physiological studies to strengthen conservation and management efforts.

Hematological parameters represent a cornerstone of wildlife health assessment, as they provide insights into oxygen transport, immune status, and the physiological impact of seasonal or reproductive strain. Red blood cell indices such as RBC, HGB, and HCT reflect oxygen-carrying capacity and aerobic performance, which may vary according to season, reproductive status, and body condition [9,10,11]. For example, post-rut males often display reduced erythrocyte indices due to the catabolic costs of reproduction, whereas females tend to maintain more stable values [12]. Leukocyte counts (WBC) provide information about immunocompetence, parasite burden, or exposure to pathogens [13,14], while platelets (PLTs), beyond their primary hemostatic role, have also been linked to inflammation and acute stress responses [15,16]. Such hematological data are therefore essential for understanding both immediate and long-term physiological adjustments in wild deer populations.

Biochemical parameters complement hematology by indicating metabolic balance, nutritional intake, and organ function. Serum urea and total protein are markers of protein turnover and nitrogen metabolism [17], glucose reflects short-term energy regulation and the metabolic costs of physical activity or reproduction [18], while cholesterol and triglycerides mirror lipid reserves and mobilization of fat stores, especially relevant during energetically demanding periods such as the rut [19,20]. In cervids, seasonal variation in these metabolites has been associated with feeding ecology, reproductive effort, and body condition [21,22]. For instance, studies have shown that nutritional condition and seasonal feeding strategies strongly influence circulating lipid and protein levels [22,23,24,25,26,27,28].

Hormonal and stress-related markers such as cortisol, testosterone, and progesterone provide additional context, as they capture physiological trade-offs between reproduction, metabolism, and stress [23,24]. Cortisol, in particular, is a widely used indicator of hypothalamic–pituitary–adrenal (HPA) axis activity in wildlife research [25,26]. Elevated cortisol has been reported in red deer during hunting or capture [27], as well as in other cervids and ungulates exposed to environmental or management stressors [29,30]. Progesterone has also been linked to reproductive status and oxidative balance in ruminants [25]. Although such endocrine markers were not measured in the present study, their mention provides a broader context, underlining the potential interactions between hormonal, hematological, and biochemical pathways in wild populations.

Despite their recognized importance, reference values for hematological and biochemical parameters remain scarce for free-ranging red deer in Eastern Europe. Most available data originate from farmed or captive individuals, or from populations in Western and Northern Europe, where ecological conditions and management differ substantially [26,28]. Furthermore, values from domestic ruminants such as sheep, goats, or cattle are often extrapolated to wild cervids, despite clear interspecific physiological differences that limit comparability [27]. In Eastern European contexts, research has historically emphasized disease surveillance rather than establishing baseline physiological parameters, leaving significant gaps in knowledge about natural variability across sex and season [21,28].

The present study was therefore designed to address these gaps. We investigated hematological and biochemical parameters in free-ranging red deer harvested during the legal hunting season in the Eastern Carpathians of Romania. We hypothesized that post-rut males would exhibit markers indicative of reproductive strain and metabolic stress, whereas females would maintain comparatively stable values across autumn and early winter. Additionally, correlation analysis was performed to highlight interdependencies among markers, providing an integrated perspective on red deer physiology under natural ecological conditions.

## 2. Materials and Methods

### 2.1. Study Area Description

The study was conducted within the FV 20 Lepșa hunting ground, located in the mountainous area of the Curvature Carpathians, Vrancea County, Romania.

The area is naturally delimited by major geographical and hydrographic features: to the north, the boundary follows OS Mănăstirea Cașin–Oituz–Sboina–Coasa Peak; to the east, it extends along the Socilor stream and then the Deju stream, up to national road DN 2D; the southern limit is defined by DN 2D road toward the village of Lepșa, continuing along the Putna and Lepșa streams; and, to the west, the boundary follows the Lepșa Ridge up to the Piatra Scrisă area (see schematic map of FV 20 Lepșa).

This region is characterized by a complex mosaic of forest and subalpine ecosystems, providing optimal conditions for the development of free-ranging red deer populations.

### 2.2. Subjects of Study

This study was conducted on a total of 40 red deer individuals, harvested from free-ranging populations inhabiting the natural environment of the FV 20 Lepșa hunting ground, Vrancea County, Romania. The biological sample was rigorously structured based on sex, season of collection, and age of the individuals in order to reduce unspecific biological variability.

The sample included the following:

- A total of 20 males: 10 collected during the autumn season (September–November), and 10 during the winter season (December–February);
- A total of 20 females: 10 collected in autumn, and 10 in winter.

The age and sex distribution of all sampled red deer is presented in Table 1. Individuals were aged between 3 and 6 years, determined primarily by dentition analysis. Age estimation was based on the eruption pattern and degree of wear of incisors, premolars, and molars: three-year-old animals show fully erupted but only slightly worn permanent teeth, whereas four- to five-year-olds exhibit moderate wear with visible changes in cusp shape and enamel grooves. By six years, molars present rounded cusps and more pronounced dentin exposure on the occlusal surface. For males, antler development was also considered, with younger adults (3–4 years) having thinner beams and fewer tines, while older individuals (5–6 years) displayed thicker antlers with more ramification. Additional criteria included overall body size and muscular condition, allowing a clear distinction between mature adults and younger subadults or senescent individuals.

Harvesting was performed exclusively during the legal hunting season, using authorized game management methods and in accordance with national legislation on sustainable wildlife management (Hunting and Game Fund Protection Law no. 407/2006, with subsequent amendments and completions).

All animals were harvested by certified personnel under conditions aimed at minimizing stress and suffering, in compliance with ethical principles for the use of animals in research. The study protocol was reviewed and approved by the Bioethics Committee of the Faculty of Food and Animal Sciences, Iași University of Life Sciences, Romania (Approval Code: 73/01.17.2024; Approval Date: 17 January 2024).

According to the provisions of Directive 2010/63/EU on the protection of animals used for scientific purposes, this study did not involve experimental procedures on live animals, as biological material was collected post-mortem from legally harvested individuals. Therefore, the present work complies with both EU regulations and national ethical requirements.

All animals included in the study were judged to be clinically healthy based on post-mortem veterinary inspection. Inclusion criteria comprised good body condition, absence of external injuries or ectoparasite infestation, and lack of gross pathological lesions in major organs. No abnormalities were reported by hunters or wardens before harvest.

### 2.3. Sample Collection

Blood samples were collected strictly post-mortem, within a maximum of 30 min after the animals had been legally harvested, in order to minimize degradation of hematological and biochemical parameters. Depending on carcass accessibility, blood was obtained either by transthoracic cardiac puncture (without full opening of the thoracic cavity) or from the jugular vein. Sampling was performed using sterile disposable needles and Vacutainer tubes under clean field conditions; no trichotomy was applied, as surgical asepsis was not required for the type of analyses conducted.

For hematological examinations, blood was collected into EDTA-K_3_ anticoagulant vacutainers, which were gently inverted to ensure proper mixing and then placed into insulated containers with cold packs to maintain ~4 °C during transport. These samples were transported under controlled temperature conditions to the laboratory and analyzed on the same day of arrival.

For biochemical analyses, blood was collected into plain Vacutainers (without anticoagulant). These tubes were maintained at 4 °C during transport and, upon arrival at the laboratory, were allowed to clot at room temperature (20–22 °C) for 30–45 min before centrifugation at 3000 rpm for 10 min. The resulting serum was transferred to sterile Eppendorf tubes, stored at 4 °C, and analyzed within 2 h.

The 30 min interval refers exclusively to the maximum time allowed between animal death and blood collection in the field. The maximum transport time from field to laboratory never exceeded 12 h, and all analyses (hematological and biochemical) were completed on the same day of collection.

### 2.4. Hematological Analysis

Hematological parameters were measured using the URIT-300 Vet Plus hematology analyzer (URIT Medical Electronic Co., Ltd., Guilin, China). The analyzer was operated in the species-specific ‘bovine’ mode, recommended by the manufacturer for cervids. Internal quality control (IQC) was performed during the analytical session using the URIT Veterinary Hematology Control (three-level commercial control material supplied by the manufacturer), covering low, normal, and high ranges for red and white blood cell counts, hemoglobin, and platelets. Control results were plotted on Levey–Jennings charts and evaluated according to Westgard rules to detect analytical shifts or trends. Calibration of the device was carried out according to the manufacturer’s recommendations, and all measurements were accepted only when the internal quality controls fell within the established reference ranges provided by the control material.

The parameters analyzed were as follows:

○ WBC (×10^3^/mm^3^): total white blood cell count;
○ RBC (×10^6^/mm^3^): total red blood cell count;
○ HGB (g/dL): hemoglobin concentration;
○ HCT (%): hematocrit;
○ PLT (×10^3^/mm^3^): total platelet count.

### 2.5. Biochemical Analysis

The biochemical determinations were conducted using the Cormay Accent 200 S automated clinical chemistry analyzer (PZ Cormay S.A., Lomianki, Poland), a multispecies-compatible device designed for use in both human and veterinary laboratories. The analyzer operates on photometric and enzymatic-kinetic principles, with each biochemical parameter measured at its specific absorbance wavelength to ensure optimal sensitivity and accuracy.

The following serum biochemical markers were quantified:

○ Glucose (mg/dL)—indicator of short-term energy metabolism and stress response;
○ Urea (mg/dL)—reflective of protein catabolism and renal function;
○ Total cholesterol (mg/dL)—related to lipid metabolism and general nutritional status;
○ Triglycerides (mg/dL)—additional marker of lipid metabolism;
○ Total protein (g/dL)—composite indicator of nutritional state, liver function, and immune status.

To ensure analytical precision and reproducibility, internal quality control procedures were implemented during the analytical session. Standardized control sera with certified analyte concentrations (provided by the manufacturer or laboratory-validated) were analyzed prior to and throughout the run, and any deviations outside the accepted ranges would have triggered recalibration or reanalysis. The use of a multispecies-compatible analyzer together with standardized quality assurance protocols ensured accurate and consistent quantification of serum biochemical parameters, providing a reliable basis for interpreting the physiological and metabolic status of free-ranging red deer under varying seasonal and ecological conditions.

### 2.6. Statistical Analysis

Statistical analysis was performed using IBM SPSS Statistics for Windows, Version 26.0 (IBM Corp., Armonk, NY, USA). All data were first tested for normality using the Shapiro–Wilk test and for homogeneity of variances using Levene’s test. Variables with a normal distribution are presented as mean ± standard deviation (SD), and group comparisons (male vs. female; autumn vs. winter) were performed using the independent samples t-test. For variables that did not follow a normal distribution, data are expressed as median and interquartile range (IQR, 25th–75th percentile), and comparisons were performed using the Mann–Whitney U test. Pearson’s correlation analysis was applied to normally distributed variables to assess associations between hematological and biochemical parameters. A *p*-value < 0.05 was considered statistically significant.

## 3. Results

### 3.1. Comparative Analysis of Hematological Parameters

For the September collection, the mean values and their dispersion are presented in Table 2. At this time of year, leukocyte counts were relatively similar between sexes, with no statistically significant difference. Erythrocyte counts (RBC) and hemoglobin concentrations (HGB) were higher in males, although these differences did not reach statistical significance (*p* > 0.05). The only parameter showing a statistically significant sex difference was hematocrit (HCT), with higher values in males (*p* = 0.0105).

For the December sampling period, the analysis revealed a similar pattern in the distribution of hematological parameters between sexes, with no significant differences observed for most variables. The exceptions were hemoglobin concentration (HGB) and platelet count (PLT), which were both significantly higher in males than in females (*p* < 0.05). Complete data for this period are summarized in Table 3.

### 3.2. The Results Regarding the Biochemical Parameters

For the group sampled in September, the mean biochemical values and inter-individual variations are presented in Table 4. No statistically significant differences were found between sexes for any of the evaluated parameters (*p* > 0.05). Total cholesterol and total proteins showed comparable values between males and females, while triglycerides, urea, and glucose tended to be slightly higher in males, without reaching statistical significance.

In December, biochemical parameters showed no statistically significant sex-related differences (Table 5). Males tended to have slightly higher mean urea and glucose levels, whereas females exhibited marginally higher cholesterol and triglyceride values. Total protein levels were comparable between sexes. All parameters remained within physiological reference ranges, indicating a uniform metabolic status across sexes during early winter.

In males, biochemical parameters measured in September and December remained broadly comparable, with no statistically significant seasonal differences (Table 6). Cholesterol, total proteins, triglycerides, urea, and glucose showed stable levels across both sampling periods, indicating consistent lipid, protein, and carbohydrate metabolism as well as balanced nitrogen turnover, despite the catabolic demands of the rut.

The overall stability of these biochemical indices indicates that, although the rut represents a period of high energetic expenditure, red deer males are able to restore metabolic homeostasis rapidly and enter winter with stable physiological profiles. This resilience likely reflects both adequate trophic resources in the Carpathian ecosystem and behavioral adaptations such as reduced activity and energy conservation strategies after the rut.

In females, biochemical parameters measured in September and December showed very similar values, with no statistically significant seasonal differences (Table 7). Cholesterol levels indicated stable lipid metabolism. Total protein concentrations also remained comparable, suggesting consistent protein turnover and dietary input between autumn and early winter. Triglyceride levels confirmed the absence of seasonal variation in fat mobilization. Urea values were nearly identical, pointing to balanced nitrogen metabolism. Likewise, glucose levels remained stable, reflecting efficient carbohydrate regulation.

Table 8 presents the Pearson correlation analysis between selected biochemical and hematological parameters—specifically Protein vs. Hemoglobin, Urea vs. Hematocrit, and Glucose vs. White Blood Cell (WBC) count—evaluated separately for male and female subjects during the months of September and December.

In September, no statistically significant correlations were observed across any of the analyzed parameter pairs, for either gender (*p* > 0.05). The correlation coefficients were weak, indicating negligible linear associations between the variables during this period.

Conversely, in December, a statistically significant negative correlation was identified between Protein and Hemoglobin in both males (r = −0.603, *p* = 0.017) and females (r = −0.606, *p* = 0.0166), suggesting a potential inverse relationship between serum protein levels and hemoglobin concentration during this month. All other associations remained statistically non-significant (*p* > 0.05).

These findings may point toward a seasonal influence on the biochemical–hematological interplay, with December showing a notable shift in the correlation between protein and hemoglobin levels.

## 4. Discussion

Total white blood cell (WBC) counts did not differ significantly between sexes, with mean values of 6.01 ± 1.49 ×10^3^/mm^3^ in males and 6.66 ± 1.76 ×10^3^/mm^3^ in females (*p* = 0.2815). Although females showed slightly higher averages, the wide overlap in ranges (3.13–8.36 in males; 3.19–9.76 in females) indicates stable immunological homeostasis across sexes. This pattern suggests that the studied animals were in good physiological condition, without evidence of infection or capture-related stress. Similar findings have been reported in free-ranging cervids, where WBC variability was more closely associated with acute stressors, parasitic load, or disease outbreaks than with sex or season [16,17,18,19]. The absence of seasonal differences in our study further supports the idea that leukocyte counts are relatively insensitive to rut-related metabolic strain or moderate hormonal changes, confirming their value as a conservative marker of immune stability in red deer populations.

Red blood cell (RBC) counts tended to be higher in males (7.17 ± 1.22 ×10^6^/mm^3^) than in females (6.40 ± 1.05 ×10^6^/mm^3^), although this difference did not reach statistical significance (*p* = 0.0745). Hemoglobin (HGB) concentrations showed a similar pattern, averaging 14.08 ± 2.08 g/dL in males versus 12.70 ± 1.80 g/dL in females (*p* = 0.0625). Taken together, these trends suggest a sex-specific erythropoietic response in males, consistent with the increased metabolic demands of the rut, when locomotor activity, reproductive competition, and territorial behavior intensify oxygen requirements. Previous studies in red deer and other cervids have reported comparable associations between erythrocyte indices, antler growth, and reproductive effort, often mediated by photoperiod-driven testosterone secretion and sustained physical stress [17,18,19]. Elevated hemoglobin has also been associated with dominance and reproductive success, underlining its role not only as a health indicator but also as a marker of ecological fitness [17]. By contrast, the relative stability of female values reflects the absence of intense reproductive strain during this period.

In December, clear sex-related differences were detected for hemoglobin (13.88 ± 2.11 g/dL in males vs. 12.04 ± 1.59 g/dL in females) and platelet counts (297.8 ± 59.9 ×10^3^/mm^3^ vs. 253.1 ± 50.9 ×10^3^/mm^3^, *p* < 0.05 for both). Hematocrit values also remained higher in males (38.2 ± 2.9% vs. 34.7 ± 3.1%), reflecting a consistent male-biased erythrocyte profile. These differences are likely driven by testosterone-induced erythropoiesis and hemoconcentration associated with reduced water and food intake during the rut, as documented in ecophysiological studies of red deer [18,19]. While such adjustments enhance short-term aerobic performance and reproductive competitiveness, persistently elevated HGB and HCT may also signal post-rut exhaustion or dehydration, with potential survival costs.

Platelet counts followed a similar trajectory, being only marginally higher in males in September but reaching statistical significance in December. Beyond their hemostatic function, platelets are increasingly recognized as markers of inflammation and stress. Elevated values in males likely reflect cumulative physical effort, microtrauma, and metabolic strain, while stress-induced splenic release of platelets has been described in cervids and mouflons under cold or high-activity conditions [16,17]. Together with erythrocyte indices, higher PLT in males may therefore represent an additional biomarker of reproductive effort and post-rut physiological strain, warranting further investigation in relation to overwintering survival.

Leukocyte (WBC) values remained stable between sexes (6.14 ± 1.70 ×10^3^/mm^3^ in males; 6.59 ± 1.91 ×10^3^/mm^3^ in females; *p* = 0.4523), confirming the maintenance of immunological homeostasis during moderate winter stress. Previous studies indicate that leukocyte counts are relatively insensitive to seasonal variation, except under infection or systemic stress [16,19]. Slightly higher values in females may reflect mild immunological activation linked to hormonal changes and cold adaptation, as reported by other authors [17].

Red blood cell (RBC) counts were comparable (6.63 ± 1.11 ×10^6^/mm^3^ in males; 6.29 ± 1.21 ×10^6^/mm^3^ in females; *p* = 0.4711). This convergence is consistent with the post-rut recovery phase, when males reduce locomotor activity and metabolic rate. Both sexes adopt energy-saving strategies, leading to reduced oxygen demand. Gaspar-López et al. (2011) reported similar trends, with gradual declines in RBC after reproduction, paralleling reduced testosterone and muscular activity [18].

Hemoglobin (HGB) concentrations were significantly higher in males (13.88 ± 2.11 g/dL) compared to females (12.04 ± 1.59 g/dL; *p* = 0.0115). This persistence of elevated male values suggests a delayed effect of autumnal erythropoiesis, a form of “hematological inertia.” Dziki-Michalska et al. (2024) proposed that high hemoglobin supports males facing thermal stress and limited resources during winter [17]. In contrast, female HGB remained stable, reflecting a more conservative physiological strategy.

Hematocrit (HCT) values also remained significantly higher in males (38.21 ± 2.94%) than in females (34.68 ± 3.11%; *p* = 0.0057). This may reflect prolonged testosterone-driven erythropoiesis, reduced water intake, or hemoconcentration due to metabolic stress. Gaspar-López et al. (2011) similarly observed persistently elevated HCT in dominant males until early winter [18]. Subclinical dehydration and metabolic strain may further contribute to this pattern.

In females, lower hematocrit values reflect both the absence of intense metabolic activity in previous months and more effective hydration or conservative plasma volume regulation. These sex-based differences are physiologically and ecologically relevant, highlighting distinct adaptive strategies during seasonal transition.

Platelet (PLT) counts were slightly higher in males (268.91 ± 51.28 ×10^3^/mm^3^) than in females (243.09 ± 48.07 ×10^3^/mm^3^), although not significant (*p* = 0.1414). This pattern mirrors September values, suggesting increased platelet mobilization in males, potentially linked to post-rut stress. In cervids, mild thrombocytosis is often interpreted as a nonspecific response to chronic stress, low-grade inflammation, or hemoconcentration [17,19]. In females, lower and more stable PLT values are consistent with the absence of reproductive strain, while the reduced sex difference in winter indicates convergence toward a common hematological profile driven by energy conservation.

In males, WBC counts (6.14 ± 1.70 ×10^3^/mm^3^; range 3.98–8.92) remained within physiological limits, confirming immunological homeostasis during moderate winter stress [16,19]. RBC averaged 6.63 ± 1.11 ×10^6^/mm^3^ (range 4.91–8.47), lower than in September, reflecting adaptation to reduced metabolic demands. Gaspar-López et al. (2011) similarly reported a post-rut decline in RBC, associated with lower testosterone and energy expenditure [18]. Hemoglobin concentrations remained high (13.88 ± 2.11 g/dL; range 10.78–16.44; *p* = 0.0115 vs. females), likely due to delayed erythropoietic stimulation or suboptimal hydration. Dziki-Michalska et al. (2024) suggested that males maintain elevated HGB post-rut as an adaptive strategy against thermal stress and energy depletion [17]. Hematocrit values were also significantly higher (38.21 ± 2.94%; range 33.74–43.40; *p* = 0.0057), supporting the hypothesis of gradual physiological recovery, with persistent hemoconcentration and residual hormone influence [18].

In females, white blood cell (WBC) counts averaged 6.59 ± 1.91 ×10^3^/mm^3^, slightly higher at the upper limit than in males, which may indicate mild immune activation in response to environmental stressors [17]. Red blood cell (RBC) values (6.29 ± 1.21 ×10^6^/mm^3^) and hematocrit (34.7 ± 3.1%) remained within physiological limits, reflecting stable hematopoietic activity and effective conservation strategies during early winter. Hemoglobin concentrations were significantly lower in females (12.04 ± 1.59 g/dL) compared to males, consistent with reduced oxygen demand and a more conservative metabolic profile [19].

Overall, the December hematological profile underscores divergent seasonal strategies between sexes: males show persistently elevated erythrocyte parameters, likely reflecting residual post-rut erythropoiesis and individual variability in rut intensity, whereas females display a more stable profile with limited fluctuations, highlighting their capacity to maintain immunological and metabolic homeostasis outside reproduction.

In females, platelet counts averaged 243.09 ± 48.07 ×10^3^/mm^3^ (range 170.36–329.71), slightly lower than in males but with wide variability, possibly related to reproductive status, microtrauma, or oxidative stress. Overall, females display a conservative hematological profile adapted to winter survival, with minimal inter-individual variation, reflecting an ecological strategy focused on resource conservation outside the reproductive season.

Cholesterol levels were slightly higher in males (119.69 ± 17.55 mg/dL) than in females (112.20 ± 17.00 mg/dL), though not significant (*p* = 0.2901). This difference may reflect residual lipid mobilization and hepatic steroid synthesis in males recovering from the rut, while lower female values suggest stable metabolism in the absence of major endocrine fluctuations [18]. Seasonal variation in cholesterol has also been linked to food availability and oxidative stress in cervids [19].

Serum urea concentrations were comparable (30.42 ± 7.18 mg/dL in males; 28.87 ± 6.49 mg/dL in females; *p* = 0.5431), indicating moderate protein catabolism during reduced winter food intake. Values fall within reported physiological ranges for wild cervids [17,20]. The slight male increase may be associated with muscle recovery post-rut but lacks pathological significance.

Blood glucose levels were nearly identical (96.93 ± 12.53 mg/dL in males; 97.63 ± 13.14 mg/dL in females; *p* = 0.8707). This stability reflects decreased locomotor activity, uniform feeding, and reliance on internal energy reserves in winter. Similar glycemic stability has been reported in both sexes as part of a seasonal overwintering strategy [17,20].

Creatinine levels were consistent across sexes (1.40 ± 0.24 mg/dL in males vs. 1.43 ± 0.21 mg/dL in females; *p* = 0.7709), confirming preserved muscle condition and normal renal function. Similar stability has been reported in cervids under non-stressful conditions, with little sex- or season-related variation [16,19]. This pattern suggests that both sexes reach a metabolic equilibrium in winter, characterized by reduced protein turnover and lower physical activity.

Total protein concentrations were slightly higher in males (6.91 ± 0.55 g/dL) than in females (6.67 ± 0.67 g/dL; *p* = 0.2946), possibly reflecting residual tissue regeneration after the rut or subtle differences in dietary intake. These values fall within physiological limits reported for European red deer [17,18]. In females, comparatively lower values may indicate more efficient protein utilization or reduced dietary input.

Albumin levels were similar between sexes (3.77 ± 0.43 g/dL in males vs. 3.72 ± 0.45 g/dL in females; *p* = 0.7763), as were A/G ratios (1.20 ± 0.21 vs. 1.23 ± 0.19; *p* = 0.6651). Given their sensitivity to hepatic function, chronic inflammation, and hydration status, the stability of these parameters reinforces the conclusion that both sexes maintained good clinical and metabolic conditions during early winter.

Albumin levels were slightly higher in males (3.77 ± 0.43 g/dL) than in females (3.72 ± 0.45 g/dL), while the albumin/globulin (A/G) ratio was similar (1.20 ± 0.21 vs. 1.23 ± 0.19; *p* = 0.7763 and *p* = 0.6651). According to Hunchak (2024), albumin fluctuations generally indicate pathological states (e.g., chronic infections, protein loss, liver disease), whereas the A/G ratio remains stable in healthy animals [19]. In this study, both parameters confirm the absence of inflammatory or hepatic dysfunction, supporting ecological and welfare assessments in red deer populations.

Serum glucose levels were nearly identical between sexes (96.93 ± 12.53 mg/dL in males; 97.63 ± 13.14 mg/dL in females; *p* = 0.8707). This uniformity reflects metabolic stabilization, as winter is characterized by reduced locomotor activity, conservative behaviors, and reliance on stored carbohydrates [17]. Stable glucose values thus confirm effective adaptation to cold conditions and the absence of metabolic stress.

Cholesterol levels were slightly higher in males (119.7 ± 17.6 mg/dL) than in females (112.2 ± 17.0 mg/dL; *p* = 0.2901), a trend also evident in September. This likely reflects persistent lipid mobilization during post-rut recovery, with ongoing energy replenishment and hepatic steroid synthesis [18]. In females, more stable cholesterol values indicate balanced lipid metabolism and consistent hepatic function. Seasonal variation in cervids has been linked to forage composition, oxidative stress, and reproductive effort [19], but in this population, lipid homeostasis appeared well maintained across sexes.

Mean urea concentrations were comparable between sexes (30.4 ± 7.2 mg/dL in males vs. 28.9 ± 6.5 mg/dL in females; *p* = 0.5431), suggesting moderate protein catabolism and efficient renal clearance. These results align with previous studies showing that urea levels in cervids during winter depend primarily on dietary protein availability rather than sex [19]. The slight male increase may reflect muscle recovery following the rut, but has no pathological significance.

Creatinine levels were consistent between sexes (1.40 ± 0.24 mg/dL in males; 1.43 ± 0.21 mg/dL in females; *p* = 0.7709), suggesting stable muscle mass and preserved renal function. This is typical of the biological resting phase (post-rut in males, anestrus in females), when diet and activity are reduced. Similar findings were reported by Ilie & Enescu (2018) and Rafaj et al. (2011) in cervids from temperate ecosystems [16,20].

Total protein concentrations were slightly higher in males (6.91 ± 0.55 g/dL) than in females (6.67 ± 0.67 g/dL; *p* = 0.2946), reflecting nutritional balance and normal hepatic function. The marginal increase in males may be linked to tissue repair after rut, while female values confirm stable adaptation and the absence of inflammatory responses [17,18].

Overall, biochemical markers indicate a shared winter metabolic equilibrium across sexes, characterized by reduced energetic demands, preserved protein metabolism, and stable hepatic and renal function. No signs of systemic stress or pathology were detected.

To further explore functional relationships among these parameters, Pearson correlation coefficients were calculated.

The correlation analysis revealed significant relationships among several hematological parameters, supporting interdependent mechanisms in erythrocyte and platelet homeostasis. The strongest association was observed between hemoglobin (HGB) and hematocrit (HCT) (r = 0.899), confirming their co-dependence in oxygen transport capacity [18,19]. Red blood cells (RBCs) were also positively correlated with both HGB (r = 0.738) and HCT (r = 0.732), consistent with the coordinated regulation of erythropoiesis. Similar patterns were reported by Barić Rafaj et al. (2011) in cervids under field conditions [16].

By contrast, white blood cell (WBC) and platelet (PLT) counts showed no significant correlations with other hematological indices, suggesting functional independence of leukocyte and thrombocyte compartments. This likely reflects their regulation by distinct physiological pathways and higher individual variability.

Among biochemical parameters, total protein (TP) and albumin (ALB) were strongly correlated (r = 0.777), as albumin represents a major serum protein fraction and both are jointly regulated by hepatic function [17,19]. A positive correlation was also found between urea and creatinine (r = 0.588), indicating coordinated nitrogen metabolism and renal excretory function [20]. Glucose and cholesterol showed no significant associations, suggesting that glycemia and lipid metabolism are influenced by independent regulatory and nutritional factors during the winter resting phase.

Sex- and season-specific correlation analyses provided additional insight into physiological regulation. In males during September, erythrocyte indices showed strong internal consistency (HGB–HCT, r = 0.897; RBC–HGB, r = 0.738; RBC–HCT, r = 0.732), pointing to synchronized erythropoiesis in response to the high energetic and oxygen demands associated with post-rut activity. In the same period, a positive correlation between glucose and albumin (r = 0.686, *p* = 0.0428) suggests an adaptive link between energy availability and hepatic protein synthesis as males prepared for the transition into winter.

In females during September, erythrocyte correlations were also evident (HGB–HCT, r = 0.782), confirming the robustness of this hematological module. However, a significant negative association between total protein and hemoglobin (r = –0.606, *p* = 0.0166) may indicate a redistribution of protein fractions toward globulins, potentially reflecting reproductive status, immune modulation, or seasonal shifts in dietary quality. Such patterns highlight the importance of considering sex-specific strategies when interpreting hematological and biochemical interrelationships in free-ranging cervids.

In December males, the erythrocyte compartment again showed strong internal coherence (HGB–HCT, r = 0.889; RBC–HGB, r = 0.783; RBC–HCT, r = 0.810), confirming synchronized regulation of oxygen transport capacity during the post-rut recovery phase [18]. Additional significant associations between total protein and albumin (r = 0.777, *p* = 0.0076) and between urea and creatinine (r = 0.588, *p* = 0.0437) reflected stable hepatic protein synthesis and renal excretory function, suggesting that males had reached a metabolic equilibrium despite residual reproductive strain [16,17,19].

In December females, the HGB–HCT correlation remained strong (r = 0.799, *p* = 0.0050), indicating consistency in erythrocyte regulation. However, a significant negative association between hemoglobin and platelet counts (r = –0.627, *p* = 0.0175) points to differential regulation of oxygen transport and thrombocyte activity during reproductive rest. In addition, the positive correlation between albumin and urea (r = 0.637, *p* = 0.0156) highlights coordinated pathways of hepatic protein synthesis and nitrogen metabolism, possibly reflecting adaptations to limited dietary input during winter [17,19].

Taken together, these correlation patterns underline the robustness of the erythrocyte module across sexes and seasons, ensuring stable oxygen-carrying capacity [18]. By contrast, biochemical variables revealed sex- and season-dependent adjustments, pointing to subtle metabolic adaptations and ecological strategies [17,19]. The presence of inverse or sex-specific associations emphasizes the influence of independent regulatory pathways and external factors such as climate, nutrition, and behavior [29]. These findings provide a basis for refining future monitoring of health, welfare, and ecological adaptability in free-ranging red deer of the Carpathians.

## 5. Study Limitations and Future Perspectives

Although the results presented provide a valuable and coherent overview of the hematological and biochemical profile of free-ranging red deer, several methodological limitations must be considered when interpreting and generalizing the conclusions.

Firstly, sampling was restricted to animals legally harvested during the hunting season, which introduces potential variability linked to the exact time of death, pre-mortem physiological responses, and the onset of post-mortem changes. Despite completing sample processing within two hours under controlled conditions, a minor pre-analytical influence on sensitive parameters cannot be entirely excluded.

Secondly, the study was limited to two seasonal time points (September and December), offering only a partial perspective on the annual physiological cycle. Key periods such as the rutting peak in late summer and the spring reproductive/early lactation period were not included, which limits the integration of endocrino-metabolic fluctuations across the full life cycle.

Thirdly, the cohort consisted exclusively of apparently healthy adult individuals, as assessed by hunters and veterinarians based on external inspection and post-mortem examination, which did not reveal visible signs of disease or parasitic infestation. Nevertheless, we cannot entirely exclude the potential influence of previous subclinical conditions on some hematological or biochemical parameters. Likewise, hormonal assays (e.g., cortisol, testosterone, thyroid hormones) were not performed, which could have provided further insight into sex- and season-specific adaptations.

Looking forward, future research should extend sampling across multiple years and additional seasons, and integrate complementary variables such as altitude, population density, habitat anthropization, and nutritional indicators. Incorporating parasitological and infectious disease diagnostics, together with hormonal, immunological, and metabolomic assessments, would allow a deeper understanding of the physiological ecology of red deer. Ultimately, such data could enhance ecological monitoring, support conservation strategies, and strengthen sustainable game management by using physiological markers as health indicators for wild cervid populations in Central and Eastern Europe.

## 6. Conclusions

The present study makes a substantial contribution to advancing the understanding of hematological and biochemical parameters in free-ranging red deer, providing a set of sex- and season-specific physiological reference values.

Through a comparative approach applied at two ecologically distinct time points—late summer (September) and early winter (December)—this research established relevant baseline values for monitoring the health status of this wild ungulate and highlighted subtle mechanisms of seasonal physiological adaptation.

Erythrocyte parameters (RBC, HGB, HCT) remained tightly correlated, reflecting a stable and functional hematopoietic system essential for maintaining oxygen homeostasis under variable environmental conditions. Significant differences between sexes, particularly in December, together with variations between the two sampling periods, indicate differentiated physiological responses linked to reproductive status, seasonal behavioral activity, and dietary changes.

Biochemical parameters reflected adaptive metabolic dynamics, with subtle sex- and season-related variations within species-specific physiological limits. Significant correlations between certain biochemical markers (urea–creatinine, total protein–albumin) support the hypothesis of well-preserved hepatic and renal function. The absence of correlations in other cases suggests functional autonomy among physiological compartments, likely influenced by extrinsic environmental factors.

From a wildlife management perspective, the establishment of hematological and biochemical reference ranges is an essential tool for assessing both individual and population-level health, enabling early detection of ecological stress, malnutrition, or early-stage pathological processes. In the broader context of ecosystem vulnerability, such physiological research becomes not only a scientific necessity but also a conservation imperative.

This study represents a key step in understanding the biological adaptability of red deer within their Carpathian range and offers a scientific basis for sustainable monitoring and protection strategies.

## Figures and Tables

**Table 1:** Distribution of sampled red deer (*Cervus elaphus*) by sex, season, and exact age.

Season	Sex	3 Years	4 Years	5 Years	6 Years	Total
Autumn	Male	2	3	3	2	10
	Female	3	2	3	2	10
Winter	Male	2	2	3	2	10
	Female	3	3	1	2	10
Total		10	10	10	10	40

**Table 2:** Hematological parameters in male and female red deer (September).

Variable	Male (*n* = 10)				Female (*n* = 10)				ANOVA
	$\bar{X}$ ± SD	Min.	Max.	Median	$\bar{X}$ ± SD	Min.	Max.	Median	*p*-Value
WBC (×10^3^/mm^3^)	6.01 ± 1.49	3.13	8.36	5.79	6.66 ± 1.76	3.19	9.76	6.47	0.2815 ^ns^
RBC (×10^6^/mm^3^)	7.17 ± 1.22	5.15	9.72	7.36	6.40 ± 1.05	4.61	8.49	6.41	0.0745 ^ns^
HGB (g/dL)	14.08 ± 2.08	9.28	16.82	13.94	12.70 ± 1.80	8.21	15.02	12.75	0.0625 ^ns^
HCT (%)	38.46 ± 3.16	32.68	44.84	38.36	35.40 ± 2.93	29.91	41.22	35.60	0.0105 *
PLT (×10^3^/mm^3^)	290.84 ± 64.84	206.96	431.43	269.79	247.27 ± 55.14	175.95	366.85	229.21	0.0574 ^ns^

* = statistically significant differences, for *p* < 0.05; ^ns^ = non-significant differences, for *p* > 0.05.

**Table 3:** Hematological parameters in male and female red deer (December).

Variable	Male (*n* = 10)				Female (*n* = 10)				ANOVA
	$\bar{X}$ ± SD	Min.	Max.	Median	$\bar{X}$ ± SD	Min.	Max.	Median	*p*-Value
WBC (×10^3^/mm^3^)	5.42 ± 1.17	3.86	8.19	5.18	6.07 ± 1.35	4.04	8.75	5.79	0.1648 ^ns^
RBC (×10^6^/mm^3^)	7.54 ± 1.01	5.38	8.77	7.89	6.88 ± 1.05	4.50	8.96	7.14	0.0912 ^ns^
HGB (g/dL)	13.97 ± 1.50	10.42	16.66	14.16	12.57 ± 1.32	9.58	15.01	12.53	0.0115 *
HCT (%)	41.34 ± 5.32	30.41	52.32	40.87	37.92 ± 4.98	27.70	47.82	37.39	0.0800 ^ns^
PLT (×10^3^/mm^3^)	297.77 ± 59.93	203.55	393.00	313.65	253.10 ± 50.88	172.84	333.46	266.54	0.0362 *

* = statistically significant differences, for *p* < 0.05; ^ns^ = non-significant differences, for *p* > 0.05.

**Table 4:** Biochemical parameters in male and female red deer (September).

Variable	Male (*n* = 10)				Female (*n* = 10)				ANOVA
	$\bar{X}$ ± SD	Min.	Max.	Median	$\bar{X}$ ± SD	Min.	Max.	Median	*p*-Value
*Cholesterol* (mg/dL)	55.51 ± 5.57	42.75	63.07	56.79	58.66 ± 5.47	48.34	66.92	59.81	0.1290 ^ns^
*Total protein* (g/dL)	63.21 ± 5.84	54.86	72.80	61.73	62.40 ± 5.55	52.87	70.66	63.16	0.7005 ^ns^
*Triglycerides* (mg/dL)	68.13 ± 10.81	51.59	90.35	70.14	65.24 ± 10.54	48.02	85.36	64.48	0.4645 ^ns^
*Urea* (mg/dL)	37.74 ± 6.86	25.13	49.38	39.07	41.26 ± 7.51	29.33	54.81	41.80	0.1913 ^ns^
*Glucose* (mg/dL)	88.18 ± 12.34	74.59	110.47	87.61	86.31 ± 12.17	72.39	109.43	86.55	0.6792 ^ns^

^ns^ = non-significant differences, for *p* > 0.05.

**Table 5:** Biochemical parameters in male and female red deer (December).

Variable	Male (*n* = 10)				Female (*n* = 10)				ANOVA
	$\bar{X}$ ± SD	Min.	Max.	Median	$\bar{X}$ ± SD	Min.	Max.	Median	*p*-Value
*Cholesterol* (mg/dL)	56.80 ± 6.20	47.17	66.33	55.39	59.26 ± 7.12	49.41	70.10	56.23	0.3200 ^ns^
*Total protein* (g/dL)	62.61 ± 5.81	54.60	73.82	60.03	61.69 ± 5.99	53.31	74.71	59.11	0.6731 ^ns^
*Triglycerides* (mg/dL)	69.10 ± 8.79	54.33	79.05	71.09	65.03 ± 8.74	50.72	74.03	69.22	0.2135 ^ns^
*Urea* (mg/dL)	38.34 ± 4.45	30.21	47.93	38.17	41.72 ± 4.95	31.82	51.32	42.02	0.0591 ^ns^
*Glucose* (mg/dL)	89.75 ± 9.18	74.92	110.75	88.43	86.58 ± 8.87	72.41	107.69	84.55	0.3436 ^ns^

^ns^ = non-significant differences, for *p* > 0.05.

**Table 6:** Biochemical parameters in male red deer (September vs. December).

Variable	Male—September (*n* = 10)				Male—December (*n* = 10)				ANOVA
	$\bar{X}$ ± SD	Min.	Max.	Median	$\bar{X}$ ± SD	Min.	Max.	Median	*p*-Value
*Cholesterol* (mg/dL)	55.51 ± 5.57	42.75	63.07	56.79	56.80 ± 6.20	47.17	66.33	55.39	0.5547 ^ns^
*Total protein* (g/dL)	63.21 ± 5.84	54.86	72.80	61.73	62.61 ± 5.81	54.60	73.82	60.03	0.7802 ^ns^
*Triglycerides* (mg/dL)	68.13 ± 10.81	51.59	90.35	70.14	69.10 ± 8.79	54.33	79.05	71.09	0.7888 ^ns^
*Urea* (mg/dL)	37.74 ± 6.86	25.13	49.38	39.07	38.34 ± 4.45	30.21	47.93	38.17	0.7801 ^ns^
*Glucose* (mg/dL)	88.18 ± 12.34	74.59	110.47	87.61	89.75 ± 9.18	74.92	110.75	88.43	0.6950 ^ns^

^ns^ = non-significant differences, for *p* > 0.05.

**Table 7:** Biochemical parameters in female red deer (September vs. December).

Variable	Female—September (*n* = 10)				Female—December (*n* = 10)				ANOVA
	$\bar{X}$ ± SD	Min.	Max.	Median	$\bar{X}$ ± SD	Min.	Max.	Median	*p*-Value
*Cholesterol* (mg/dL)	58.66 ± 5.47	48.34	66.92	59.81	59.26 ± 7.12	49.41	70.10	56.23	0.7970 ^ns^
*Total protein* (g/dL)	62.40 ± 5.55	52.87	70.66	63.16	61.69 ± 5.99	53.31	74.71	59.11	0.7387 ^ns^
*Triglycerides* (mg/dL)	65.24 ± 10.54	48.02	85.36	64.48	65.03 ± 8.74	50.72	74.03	69.22	0.9530 ^ns^
*Urea* (mg/dL)	41.26 ± 7.51	29.33	54.81	41.80	41.72 ± 4.95	31.82	51.32	42.02	0.8441 ^ns^
*Glucose* (mg/dL)	86.31 ± 12.17	72.39	109.43	86.55	86.58 ± 8.87	72.41	107.69	84.55	0.9452 ^ns^

^ns^ = non-significant differences, for *p* > 0.05.

**Table 8:** Pearson’s correlation between selected biochemical and hematological parameters in male and female red deer (September and December).

Month	Group	Correlated Parameters	Correlation Coefficient (r)	*p*-Value	Statistical Significance
September	Males	Protein vs. Hemoglobin	−0.435	0.105	Not significant (*p* > 0.05)
September	Females	Protein vs. Hemoglobin	−0.189	0.499	Not significant (*p* > 0.05)
September	Males	Urea vs. Hematocrit	0.055	0.846	Not significant (*p* > 0.05)
September	Females	Urea vs. Hematocrit	0.045	0.874	Not significant (*p* > 0.05)
September	Males	Glucose vs. WBC	0.007	0.982	Not significant (*p* > 0.05)
September	Females	Glucose vs. WBC	−0.003	0.880	Not significant (*p* > 0.05)
December	Males	Protein vs. Hemoglobin	−0.603	0.017	Significant (0.01 < *p* < 0.05)
December	Females	Protein vs. Hemoglobin	−0.606	0.0166	Significant (0.01 < *p* < 0.05)
December	Males	Urea vs. Hematocrit	−0.151	0.514	Not significant (*p* > 0.05)
December	Females	Urea vs. Hematocrit	−0.090	0.749	Not significant (*p* > 0.05)
December	Males	Glucose vs. WBC	−0.122	0.665	Not significant (*p* > 0.05)
December	Females	Glucose vs. WBC	−0.003	0.916	Not significant (*p* > 0.05)

## Data Availability

The original contributions presented in this study are included in the article material. Further inquiries can be directed to the corresponding author.
